# Gait deviations of patients with ruptured anterior cruciate ligament: a cross-sectional gait analysis study on male patients

**DOI:** 10.1186/s43019-021-00128-w

**Published:** 2021-12-24

**Authors:** Jay Hoon Park, Min-Ho Choi, Joonhee Lee, Hyuk-Soo Han, Myung Chul Lee, Du Hyun Ro

**Affiliations:** 1 Department of Orthopaedic Surgery, Seoul National University Hospital, 101 Daehak-ro, Jongno-gu, Seoul, 110-744 Korea; 2 Seoul National University College of Medicine, Seoul, South Korea

## Background

Anterior cruciate ligament (ACL) is the primary restraint of anteroposterior instability and the secondary restraint of rotational instability. Rupture of ACL results in knee instability [1, 2], and the instability can affect kinematics and kinetics of the knee joint [3–12]. Kinematics describe the different angulation of the knee joint, whereas kinetics explain the change in knee joint moment.

Gait deviation in patients with ACL rupture was first reported by Berchuck et al. in 1990 [6]. They proposed a “quadriceps avoidance gait,” wherein patients with ACL rupture walked with reduced quadriceps activation to decrease anterior tibial translation. It is the most-cited gait adaptation mechanism of ACL rupture patients. However, subsequent research raised counterarguments [5–11, 13]. Firstly, several researchers noted the absence the of quadriceps avoidance gait pattern in their group of patients [7, 9, 12, 13]. Secondly, knee was extended at the initial stance phase, as opposed to the mid-stance phase reported by Berchuck [8, 10, 14, 15]. It is worthwhile noting that the methodology among studies is inconsistent. Patient selection criteria, gait examination timing, and diagnostic method varied among the studies, and conclusions were inconsistent. Currently, the presence of quadriceps avoidance gait as well as the gait adaptation mechanisms after ACL rupture is inconsistent across the literature [3–13].

Information regarding the change in gait pattern may provide further insight into neuromuscular strategies of patients with ACL rupture, in addition to shedding light on the pathophysiology, by which arthritis tends to develop in ACL-deficient knees. Therefore, in the present study, we performed a comprehensive analysis of knee biomechanics using principal component analysis (PCA). Unlike simple comparison of maximal or minimal values, PCA can analyze a large matrix of data without loss of information.

In this study, we tested the hypothesis that kinematic and kinetic deviations of ACL rupture exist, and that these deviations may act as a stabilization strategy. The purpose of this study was to find kinematic and kinetic deviations of ACL-ruptured knees by comparing them with their contralateral uninjured knee in the axial and sagittal planes.

## Methods

### Participants

**Fig. 1:**
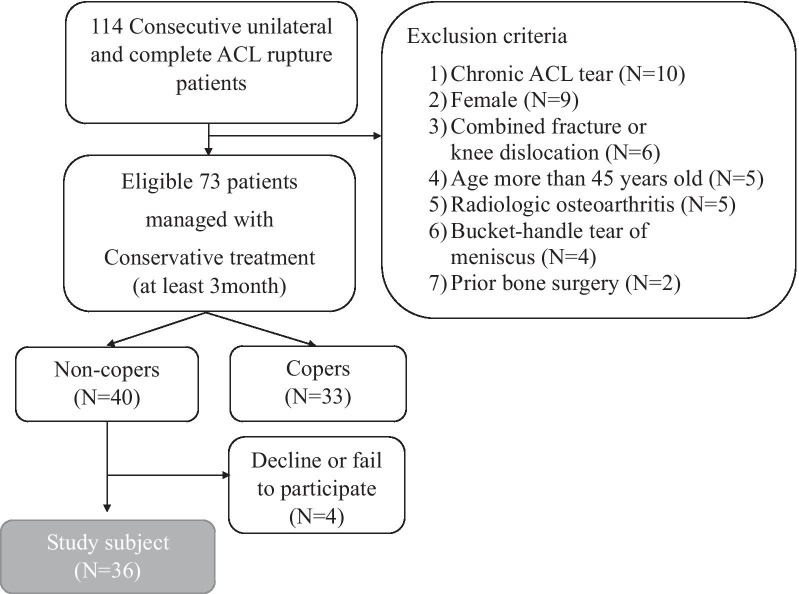
Study design and eligibility criteria. Thirty-six patients were included in this cross-sectional study

This cross-sectional gait analysis study was conducted on 114 patients with complete unilateral ACL rupture (Fig. 1). ACL rupture was diagnosed clinically and confirmed by magnetic resonance imaging (MRI). MRI criteria applied to assess ACL rupture were fiber discontinuity, abnormal orientation of ACL with respect the Blumensaat’s line, and empty notch sign. To minimize possible confounding effects [3, 4, 16], patients meeting the following criteria were excluded: (1) chronic ACL tear (more than 6 months at first visit) (*N* = 10); (2) female sex (because of sex differences in gait properties [17–20]) (*N* = 9); (3) combined fracture or knee dislocation (*N* = 6); (4) aged more than 45 years (*N* = 5); (5) evidence of radiologic osteoarthritis (Kellegren–Lawrence grade II or more) (*N* = 5); (6) combined bucket-handle tear of meniscus (*N* = 4); and (7) any prior surgery in the lower extremity (*N* = 2). After application of the exclusion criteria, 73 eligible patients remained. Initially, the patients were treated conservatively through physical therapy and strengthening exercise. Since the patients were transferred to our hospital after conservative treatment at another institution, the duration of conservative treatment varied in patients. After at least 3 months of conservative treatment, more than half of the patients (40 patients) complained of instability or giving-way, even though they had significantly reduced their activity level prior to injury. We defined these patients as “non-copers” in this paper and included them in the analysis [21]. The non-copers also underwent ACL reconstruction surgery after this study. Of the non-copers, four declined or failed to participate in the study. Therefore, 36 males with unilateral ACL rupture, who were functionally classified as non-copers, participated in this study. The mean subject age was 27.1 years (± 7.0, range 19–45 years), the mean height was 172 (± 7.2) cm, and the mean weight was 71.5 (± 11.2) kg. The average range of motion (ROM) prior to gait laboratory analysis was 138.7° (± 15.8°). The average Lysholm score was 67.7 (± 12.7). The pivot shift test results were Gr I for 11 patients, Gr II for 17 patients, and Gr III for 6 patients. The Lachmann test results were Gr I for 8 patients, Gr II for 22 patients, and Gr III for 6 patients.

### Data collection

Gait data were measured in the Human Motion Analysis Lab at our institution. The average time interval between the injury and gait measurement was 4 months (range 3–8 months). Before gait measurements, subjects underwent conservative treatment until they met the following criteria: minimal knee effusion, no knee extension deficit, minimal pain in the injured limb with walking, and no visually identifiable gait impairments. The average pain score assessed on a numeric rating scale at the time of gait analysis was 1.2 (± 0.8). Therefore, we could exclude any acute effects of knee trauma on the basis of the criteria above. Patients were asked to perform 5 min of easy walking to warm up. After warming up, reflective markers from a Helen Hayes marker set were placed on each subject’s body [22]. Patients were asked to walk at their usual speed along a 9-m track.

In our gait analysis, we defined kinematic control as the different angulation of the knee joint and kinetic control as the change of moment by the action of the quadriceps. Motion (kinematic) data were acquired at a sample rate of 120 Hz using 12 charge-coupled device cameras equipped with a three-dimensional optical motion capture system (Motion Analysis, Santa Rosa, USA). Ground reaction force (kinetic) data were acquired at a sampling rate of 1200 Hz using three AMTI (Advanced Mechanical Technology Inc., Watertown, MA, USA) force plates. The kinetic data were then normalized by height and weight (% body weight × height) [23].

We used Eva Real-Time software (Motion Analysis, Santa Rosa, USA), Microsoft Excel 2016 (Microsoft, Redmond, USA), and MATLAB R2017a (Mathworks, Natick, MA, USA) for real-time motion capture, post-processing, and marker data tracking. The average of three representative strides from five or six separate trials was used for the analysis of each session. Two kinematic gait parameters (knee flexion angle, knee rotation angle) and two kinetic parameters [internal knee extension moment (KEM), knee rotation moment] were measured.

### Statistical analysis

Peak (maximum) kinematic and kinetic data of ACL-ruptured and uninjured limbs were compared using paired Student’s *t*-tests. Four gait parameters, knee flexion angle, knee rotation angle, internal knee extension moment (KEM), and knee rotation moment were processed via principal component analysis (PCA). PCA was performed via two steps. First, features were extracted from each gait parameter. Second, each feature was scored for each subject. Each gait parameter consisted of an *n* × 101 data matrix, where *n* rows represent the number of cases and 101 columns represent standardized 101 timepoints. PCA processes this gait parameter matrix with an orthogonal transformation and extracts the data into a set of gait features that are linearly uncorrelated (principal components, PCs). These transformations allow the major features (PCs) of each gait parameter to be recognized. PCs were selected using a 90% trace criterion, and seven features were extracted from two gait parameters (i.e., KEM and knee flexion angle). The next step was the scoring of the extracted features. This was obtained by standardizing individual contributions to the extracted features [24]. In the case of a high score, the standardized individual contribution shows the same direction as the extracted feature. Conversely, when the score is low or negative, the contribution is applied the opposite direction. The PC score is a standardized score (mean = 0, standard deviation = 1) that can be compared with other subjects or among the features. The standardized mean difference (SMD) of each of the PC scores was compared between the ACL-ruptured and uninjured knees. The feature that showed the highest SMD of the PC score was then investigated.

The PC scores of ACL-ruptured and uninjured limbs were compared using paired Student’s *t*-tests. The normality of each PC score was assessed with the Kolmogorov–Smirnov test. All statistical analyses were performed using SPSS 19.0.1 for Windows (SPSS Inc., Chicago, IL, USA). *P*-values < 0.05 were considered to indicate statistical significance.

## Results

Notable kinematic patterns in terms of knee flexion angle and kinetic patterns of decreased KEM were observed during phases of the gait cycle. Analysis of the association between the kinematic and kinetic patterns at different gait phases revealed two distinctive kinematic strategies adopted by the ACL-injured knee.

**Fig. 2:**
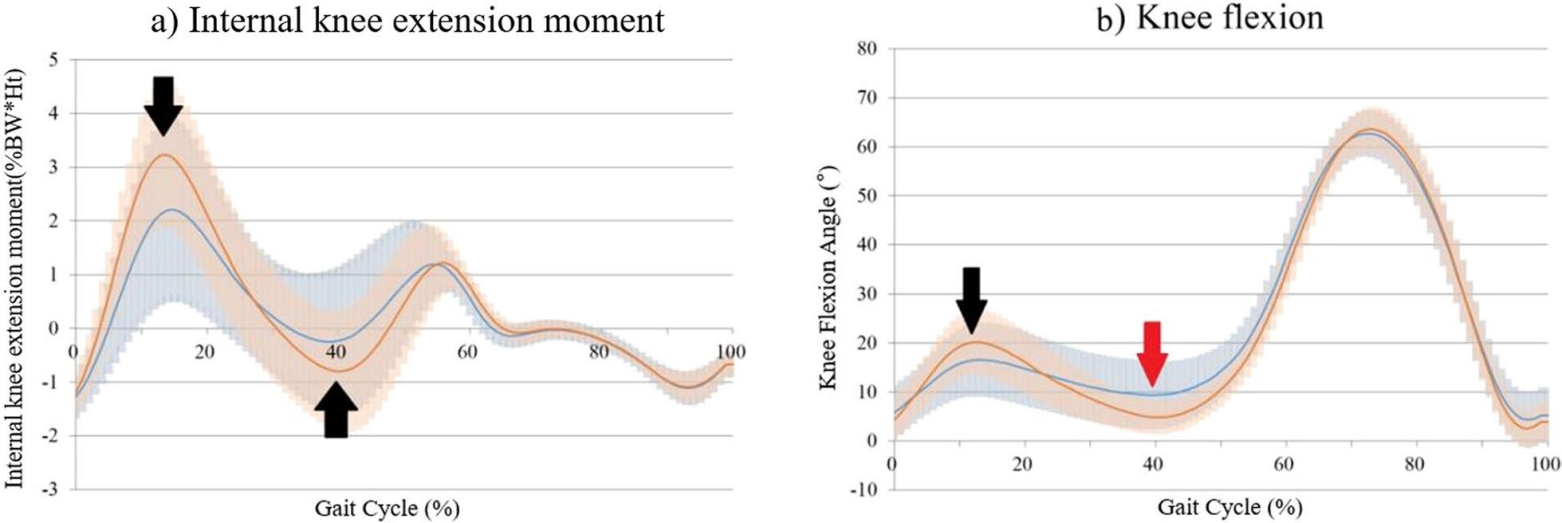
Kinetics and kinematics of the knee joint in the sagittal and axial planes. The blue curve indicates the ACL-ruptured limb, and the red curve indicates the contralateral uninjured limb. The shaded region represents mean ± one standard deviation. Table 1 presents a statistical analysis of the graph. **a** Knee extension moment. The knee extension moment peak value and amplitude were both smaller in the ACL-ruptured limb (black arrow). **b** Knee flexion angle. The ACL-ruptured knee showed extension at the IDS phase (black arrow) and more flexion from the SLS to the TDS phase (red arrow)

Two distinctive kinematic patterns were observed in knee flexion angle (Table 1, Fig. 2b). Patients extended their ACL-ruptured knee more at the initial double-limb stance (IDS) phase and then flexed more during the single-limb stance (SLS) to the terminal double-limb stance (TDS) phase (both *P* < 0.001) compared with the contralateral uninjured knee (black and red arrows in Fig. 2b).

**Table 1:** Principal component analysis of kinetics and kinematics

Gait measure	PC	Feature	% of variance	SMD	*P*-value*
			(cumulative)	(*N* = 36)	
(a) Knee extension moment (KEM)	PC1	Overall magnitude	68.5	0.15	0.542
	PC2	Amplitude of moment	89.8	1.02	** < 0.001**
	PC3	Phase shift	95.6	0.11	0.483
	Interpretation: less amplitude of moment (of ACL ruptured limb)				
(b) Knee flexion angle	PC1	Overall flexion angle	56.8	0.3	0.069
	PC2	Flexion at swing phase	75.9	0.26	0.157
	PC3	Flexion at SLS to TDS phase	88.2	0.87	** < 0.001**
	PC4	Flexion at IDS phase	94.8	0.76	** < 0.001**
	Interpretation: extension at IDS phase and more flexion at SLS to TDS phase(of ACL ruptured limb)				

*PC* principal component, *SMD* standardized mean difference, *IDS* initial double-limb support, *SLS* single-limb support, *TDS* terminal double-limb support, *IR* internal rotation

^*^Comparisons were made with paired *t*-test. Bold face indicates statistical significance

The most significant kinetic difference between the ACL-ruptured and uninjured knees was the peak-to-peak amplitude (i.e., difference between peak and trough) of KEM (Table 1, Fig. 2a). It was smaller in the ACL-ruptured knees, and the SMD was 1.02 (*P* < 0.001). The peak KEM in the ACL-ruptured knees was 27% smaller than that of the uninjured knees (*P* < 0.002, ACL-ruptured: 2.5 (%Body weight * Height), uninjured: 3.4 (%Body weight * Height)).

**Fig. 3:**
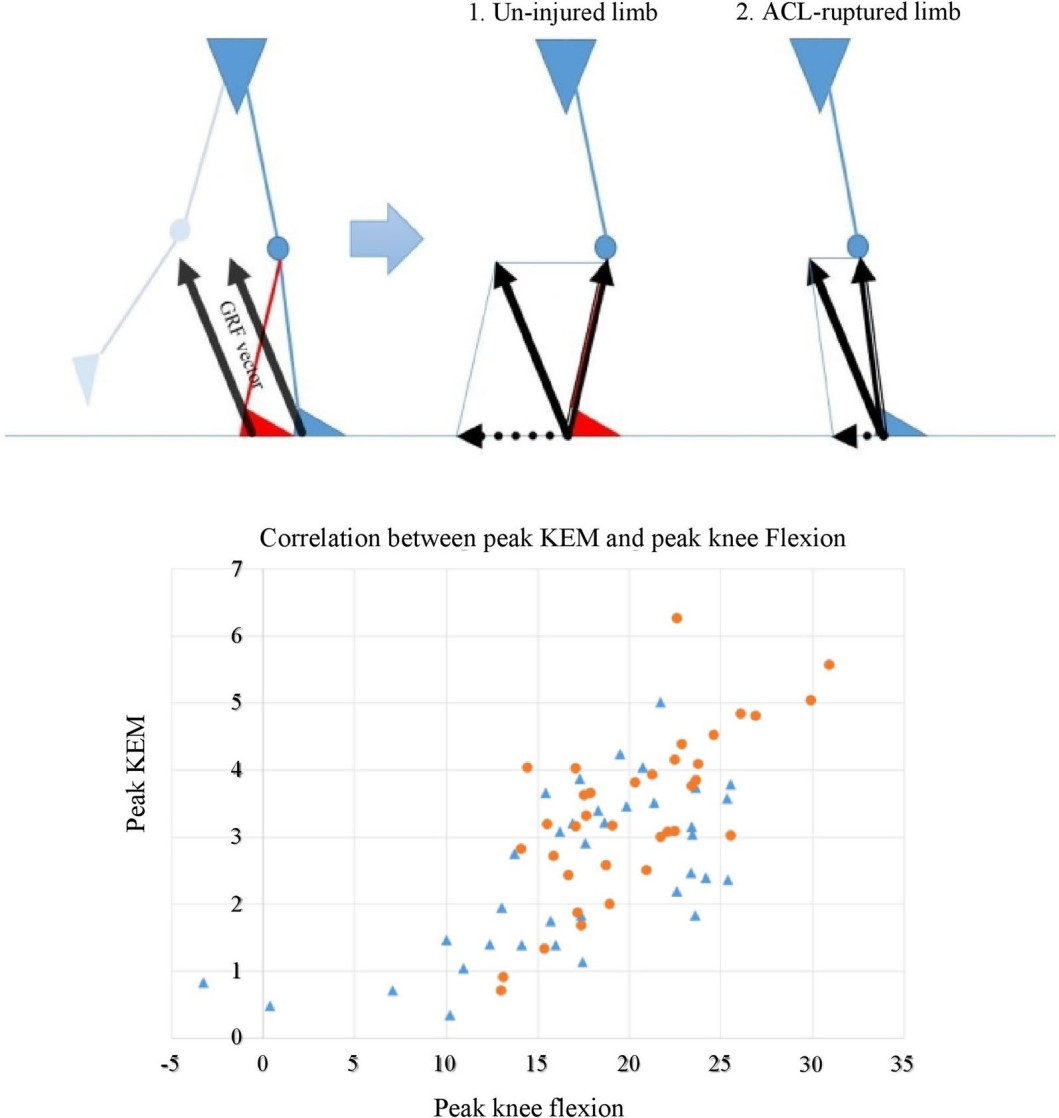
Schematic representations of knee extension moment (KEM) and knee flexion angle at the initial double-limb stance (IDS) phase. During this phase, ground reaction force (GRF) is generated for the repulsive body weight force (black arrow). The GRF can be divided into an axial vector (double arrow) and a transverse vector (dotted arrow). The axial vector runs parallel to the tibia and acts as a compressive force to the tibiofemoral joint. The transverse vector runs parallel to the ground and acts as a knee flexion force (counter to the knee extension moment by the quadriceps). The ACL-ruptured knee can be unstable during this phase, so patients try to reduce the transverse vector by extending their knee (note the difference in knee flexion angle). Instead, the tibiofemoral joint axial force can be increased. The graph shows the correlations between peak KEM and peak knee flexion at the IDS phase. The blue triangle represents the ACL-ruptured limb, and the orange circle represents the uninjured limb. Note the strong correlation between the two variables (Pearson *r* = 0.694, *P* < 0.001). Linear regression analysis showed that the adjusted *R*^2^ value of the first strategy was 0.475

**Fig. 4:**
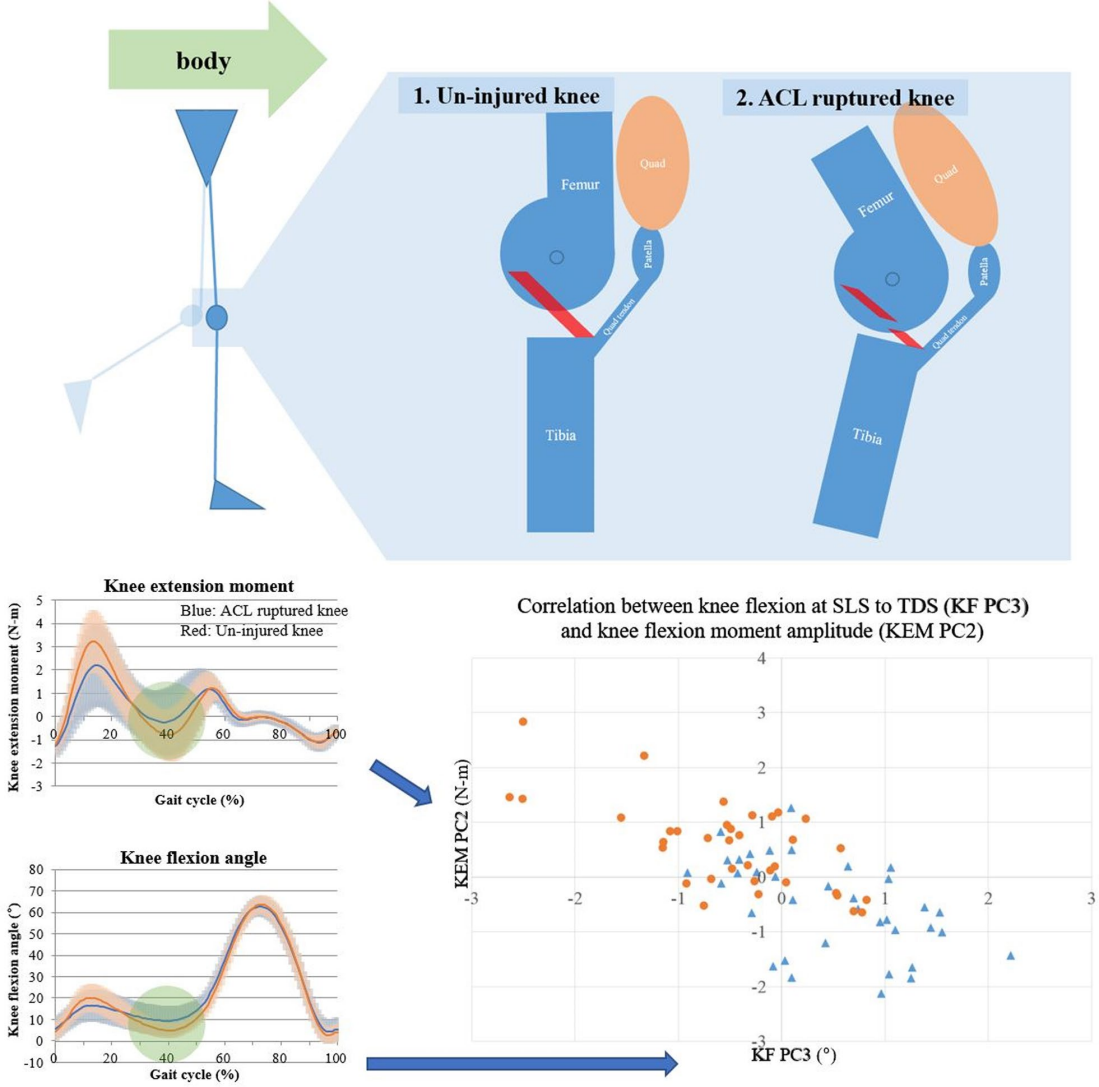
Schematic representations of knee extension moment (KEM) and knee flexion angle during progression from the single-limb stance (SLS) to the terminal double-limb stance (TDS) phase. During this phase, the knee joint is more flexed in ACL-ruptured knees. Extended knees can be unstable during this phase because the KEM rapidly becomes negative (see the green KEM circle). Patients try to decrease the speed of the KEM changes by flexing their ACL-ruptured knee. This strategy has previously been described as the “quadriceps avoidance or stiffening strategy.” The lower right graph shows the correlation between KF PC3 (knee flexion principal component 2) and KEM PC2. KF PC3 represents the knee flexion angle during progression from the SLS to the TDS phase. KEM PC2 represents the KEM amplitude. The blue triangle represents the ACL-ruptured limb, and the orange circle represents the uninjured limb. Linear regression analysis showed that the adjusted R^2^ value of the second strategy was 0.497

We subsequently investigated the association between kinematic and kinetic parameters. At the IDS phase (i.e., loading phase), the peak knee flexion angles of the ACL-ruptured knees and uninjured knees were 17.1° and 20.2°, respectively (*P* < 0.001). This angle was positively correlated with the peak KEM (Pearson *r* = 0.694, *P* < 0.001) (Fig. 3). In addition, knee flexion angle at SLS to TDS (i.e., KF PC3) was higher in ACL-ruptured knees (*P* < 0.001) and was negatively correlated with the KEM amplitude (i.e., KEM PC2) (Pearson *r* = −0.710, *P* < 0.001) (Fig. 4).

Cumulatively, these data indicate that patients adopted two distinctive kinematic strategies to reduce KEM peak and amplitude of their ACL-ruptured knee. In the first strategy, patients extended their ACL-ruptured knee 3.1° more at the IDS phase to reduce the peak KEM. In the second strategy, patients flexed their ACL-ruptured knee at SLS to TDS phase to reduce the KEM amplitude. Linear regression analysis showed that the adjusted R^2^ of the first strategy was 0.475 and that of the second strategy was 0.497 (Table 2).

**Table 2:** Regression model for kinematic strategy

Regression models	Peak KEM		
	*ß* ± SE*	*P*-value	*R*^2^adj†
(1) Knee flexion at IDS	0.152 ± 0.019	< 0.001	0.475

	Amplitude of KEM		
	*ß* ± SE*	*P*-value	*R*^2^adj†
(2) Knee flexion at SLS to TDS	−0.731 ± 0.087	< 0.001	0.497

^*^Values are given as the *ß* (standardized regression coefficient) and SE (standard error)

^†^*R*^2^adj = % variance explained by each variable

## Discussion

The most important findings of this study are that we found two distinct kinematic deviations on ACL-ruptured knee associated with the peak and amplitude of KEM, respectively. At IDS phase of the gait cycle, kinematic strategy to extend the knee contributed to the reduction of peak KEM, showing consistency with the quadriceps avoidance strategy. At SLS to TDS phase, kinematic strategy to flex the knee leads to reduced KEM amplitude, reflecting the stiffening strategy. Our findings suggest that kinematic control of knee joint is an important gait deviation mechanism in patients with ACL rupture.

. To our knowledge, this is the first study to describe two distinctive kinematic controls associated with kinetics in patients with ACL rupture. Berchuck et al. found that the KEM was decreased and sometimes even reversed in the mid-stance walking phase of patients with ACL rupture, terming this phenomenon “quadriceps avoidance gait” [6]. However, subsequent studies did not yield consistent results. Studies by Georgoulis et al. (and others) found no difference in sagittal-plane kinematics, while studies by Hurd et al. (and others) found significant KEM reduction [3–13]. More recent reports with delicate study designs found that patients with ACL rupture had a reduced KEM, although the KEM was still greater than zero and was not reversed, as in the original article [5, 8, 13]. We noticed that the studies that did not observe the reduction of KEM used different patient selection criteria and/or different gait examination timing. For example, Georgoulis and colleagues performed the gait examinations on average at 7.6 ± 4.3 weeks after ACL rupture and did not classify patients as copers or non-copers [12]. Furthermore, Berchuck et al. found normal biphasic patterns in 25% of the analyzed patients, suggesting that pattern results may vary according to patient selection criteria [6]. In the present work, we excluded females, copers, and acute or chronic ACL rupture to minimize these possible confounding effects. As described above, gait features differ according to sex [17–20]. An acute ACL rupture can result in antalgic gait, whereas chronic ACL rupture can result in arthritic gait features [3, 12]. Proper control and selection of the patient group are very important. Further studies analyzed the kinetic patterns of quadriceps avoidance gait by examining muscle strength and electromyography (EMG) [5, 16]. However, kinematic control has not been widely studied, even though this type of control is one of the main means of neuromuscular control in patients with ACL rupture.

To understand the gait deviation of patients with ACL rupture, one must examine the mechanism involved when the internal KEM decreases. The most common interpretation is direct inhibition of the quadriceps femoris [5, 16]. The internal KEM is generated by eccentric contraction of the quadriceps with a moment opposite to the external knee flexion moment (KFM), which acts as an external flexion force in the loading phase. The KEM has the same size as the KFM, but the opposite mechanical balance. During gait, the KEM can act as an anterior translation force for the proximal tibia; thus, the quadriceps is unconsciously suppressed in the patients with ACL rupture [5, 6, 16]. In support of this hypothesis, studies using EMG have shown that quadriceps muscle activity is suppressed in patients with ACL rupture [15, 16]. In addition, increased hamstring activity is associated with this suppression. This increase in muscle activity is referred to as muscle coactivation; both phenomena are considered major neuromuscular adaptations in patients with ACL rupture [5, 14–16, 25].

Unlike the study by Berchuck et al. that described the quadriceps avoidance pattern at the mid-stance phase, our study, as well as previous studies, reported knee extensions at the IDS phase [8, 10, 14, 15]. We investigated the relationship between peak KEM and peak knee flexion angle in the IDS phase and found a strong linear relationship (Pearson *r* = 0.694, *P* < 0.001). The results showed that knee extension in the IDS phase reduces the KEM. Extension of the knee in the IDS phase has been observed in previous studies but was not previously interpreted mathematically as in the present study [3, 5, 10]. These results suggest that both kinematic control and kinetic control may be associated with the gait of patients with ACL rupture. In the uninjured leg, knee flexion occurs with quadriceps eccentric contraction to reduce GRF during the IDS phase. However, an ACL-ruptured leg creates knee extension at the IDS phase to minimize the use of the quadriceps, and consequently reduces KEM. When the knee is further extended, the transverse vector decreases, reducing the force applied to the anteroposterior direction of the tibia [6] (Fig. 3). However, this reduction is expected to increase the GRF distribution in the axial direction (while decreasing the transverse vector). This increased GRF distribution may increase the impact on the tibiofemoral (TF) joint and may also contribute to the development of TF arthritis or subsequent meniscus injury after ACL rupture [10, 11]. In conclusion, this study is meaningful in interpreting the quadriceps avoidance gait at the IDS phase from the viewpoint of kinematic control. This phenomenon likely corresponds to a centrally controlled mechanism of the knee joint [26]. A study in a rat model showed that the central nervous system (CNS) regulates muscle activation to reduce the load within the joint [27]. We think that similar regulation occurs centrally in ACL-injured knees in terms of kinematic control of the joint. This strategy could be a coordinated way to reduce peak KEM in the early stages through feed-forward signaling at the IDS phase by kinematic control.

After the IDS phase, the walking strategy from the SLS to the TDS phase was similar to the stiffening strategy described by Hurd et al [5, 6, 8, 15]. However, the knee stiffening strategy in the SLS to TDS phases seems to affect the amplitude rather than the KEM peak value. When the two walking strategies were modeled by regression analysis (Table 2), the adjusted *R*^2^ values were 0.475 and 0.497. These correlations could account for significant portions of the KEM peak and amplitude. The rest of the KEM peak and amplitude are likely to be due to direct inhibition or muscle coactivation, which were not included in this study. The correlation has been observed by others [5].

This study has some limitations. First, participants were restricted to non-coping men. This selection limits the degree to which the results of this study can be applied to other groups. Women are known to have greater rotational laxity than men; therefore, the results may be different in women [17–20]. However, since men and women have different gait patterns and skeletal alignments, analyzing men and women together without controls could make the results harder to interpret [17–20]. Future research should focus on women. Second, only non-copers were tested. Nonetheless, analysis of the gait pattern of copers is not as important as that of non-copers at present. Moreover, analysis that fails to discriminate non-copers from copers could lead to inadequate conclusions. Third, the gait and clinical tests were performed between 3 and 8 months after the injury, meaning that pain and stiffness may have affected the gait. As a retrospective study, the patients arrived at the institution at different timepoints following the injury. The result was that the conservative treatment at other hospitals prior to the first visit varied. However, before gait measurements, each participant was verified to have minimal knee effusion, no knee extension deficits, minimal pain in the injured limb with walking, and no visually identifiable gait impairments. These criteria were applied to minimize the effects of pain and stiffness. The average pain numeric rating was 1.2 ± 0.8, and the average range of motion (ROM) prior to gait analysis in the laboratory was 138.7 ± 15.8°. However, the gait patterns of patients with acute or chronic ACL ligament rupture may be different; therefore, further studies are needed [3, 12]. This study does not have healthy subjects as a control; instead, contralateral uninjured limb was used. ACL rupture can affect the gait pattern of the opposite limb as well. Having a control group of healthy individuals will enhance our understanding of the gait deviation in patients with ACL injury. In the future, we hope to expand the studies with healthy subjects included. Lastly, quadriceps atrophy may be present in patients with ACL injury. No subject had severe observable quadriceps atrophy, especially because the time between the injury and the gait study was short. Nevertheless, quantitative assessment of quadriceps atrophy would allow the assessment of possible confounding effects that the atrophy may have on the gait analysis.

## Conclusions

Patients showed two distinct kinematic deviations to reduce the KEM peak and amplitude of their ACL-ruptured knee. This suggests that kinematic control of knee joint is an important gait deviation mechanism of patients with ACL rupture. Our findings should be taken into consideration when treating patients with ACL ruptured conservatively.

## Data Availability

The datasets used and/or analyzed during the current study are available from the corresponding author on reasonable request.

## Abbreviations

ACL: Anterior cruciate ligament
EMG: Electromyography
GRF: Ground reaction force
IDS: Initial double limb stance
KEM: Knee extension moment
KFM: Knee flexion moment
MRI: Magnetic resonance imaging
PC: Principal component
PCA: Principal component analysis
ROM: Range of motion
SLS: Single-limb stance
SMD: Standardized mean deviation
TDS: Terminal double-limb stance
TF: Tibiofemoral
